# Sodium selectivity of Reissner's membrane epithelial cells

**DOI:** 10.1186/1472-6793-11-4

**Published:** 2011-02-01

**Authors:** Muneharu Yamazaki, Kyunghee X Kim, Daniel C Marcus

**Affiliations:** 1 Cellular Biophysics Laboratory, Dept. Anatomy & Physiology, Kansas State University, Manhattan, KS 66506 USA; 2 Dept. of Otolaryngology-Head and Neck Surgery, Tohoku University Graduate School of Medicine, Sendai, 980-8574 Japan

## Abstract

**Background:**

Sodium absorption by Reissner's membrane is thought to contribute to the homeostasis of the volume of cochlear endolymph. It was previously shown that the absorptive transepithelial current was blocked by amiloride and benzamil. The most commonly-observed target of these drugs is the epithelial sodium channel (ENaC), which is composed of the three subunits α-,β- and γ-ENaC. However, other less-selective cation channels have also been observed to be sensitive to benzamil and amiloride. The aim of this study was to determine whether Reissner's membrane epithelial cells could support parasensory K^+ ^absorption via amiloride- and benzamil-sensitive electrogenic pathways.

**Results:**

We determined the molecular and functional expression of candidate cation channels with gene array (GEO GSE6196), RT-PCR, and whole-cell patch clamp. Transcript expression analysis of Reissner's membrane detected no amiloride-sensitive acid-sensing ion channels (ASIC1a, ASIC2a, ASIC2b) nor amiloride-sensitive cyclic-nucleotide gated channels (CNGA1, CNGA2, CNGA4, CNGB3). By contrast, α-,β- and γ-ENaC were all previously reported as present in Reissner's membrane. The selectivity of the benzamil-sensitive cation currents was observed in whole-cell patch clamp recordings under Cl^-^-free conditions where cations were the only permeant species. The currents were carried by Na^+ ^but not K^+^, and the permeability of Li^+ ^was greater than that of Na^+ ^in Reissner's membrane. Complete replacement of bath Na^+ ^with the inpermeable cation NMDG^+ ^led to the same inward current as with benzamil in a Na^+ ^bath.

**Conclusions:**

These results are consistent with the amiloride/benzamil-sensitive absorptive flux of Reissner's membrane mediated by a highly Na^+^-selective channel that has several key characteristics in common with αβγ-ENaC. The amiloride-sensitive pathway therefore absorbs only Na^+ ^in this epithelium and does not provide a parasensory K^+ ^efflux route from scala media.

## Background

The inner ear has absorptive pathways for both Na^+ ^and K^+ ^that contribute to the homeostasis of the composition of endolymph, the luminal fluid. The regulation of the ion composition of endolymph is essential for normal hearing [1,2]. Transepithelial K^+ ^efflux through the sensory hair cells in the cochlea is responsible for detection of sound. Parasensory K^+ ^absorption through other cell types is needed to compensate for changes in sensory cell K^+ ^flux due to changes in levels of stimulation from acoustic inputs. The cochlear outer sulcus is an epithelial domain known to participate in absorption of both K^+ ^and Na^+ ^[3].

Absorptive mechanisms are needed to remove Na^+ ^from endolymph in order to maintain osmotic balance, to prevent loading of sensory hair cells with Na^+ ^and to maintain functional physical properties of the tectorial membrane. Na^+^, like K^+^, is absorbed via nonselective cation channels in the apical membranes of outer sulcus cells. In addition, Na^+ ^appears to be absorbed via an amiloride-sensitive pathway in Reissner's membrane (RM) of the cochlea.

The transepithelial current across RM was shown to be inhibited by amiloride and its analog, benzamil [4,5]. The most commonly-observed target of these drugs is the epithelial sodium channel (ENaC), which is usually composed of the three subunits α-,β- and γ-ENaC. However, other combinations of ENaC subunits and other cation channels have also been observed to be sensitive to amiloride and benzamil. Further, those channels are not as selectively permeable to Na^+ ^over K^+ ^and would therefore provide a potential pathway for parasensory K^+^-absorption.

In view of the high luminal concentration of K^+ ^in the inner ear (ca. 150 mM) and the importance of K^+ ^efflux pathways for endolymph homeostasis, we investigated whether RM epithelial cells could support parasensory K^+ ^absorption via amiloride-sensitive electrogenic pathways. The results show that acutely isolated RM has a highly Na^+^-selective transport pathway, without detectable contributions from K^+^. The processes studied have several properties of the classical ENaC channel including inhibition by amiloride and benzamil, high selectivity for Na^+ ^over K^+ ^and a higher permeability to Li^+ ^over Na^+^.

## Results

We have shown in previous studies that Reissner's membrane in mouse and gerbil absorbs Na^+ ^from the cochlear lumen by electrogenic transepithelial transport, which was apparently mediated by apical ENaC, basolateral Na^+^,K^+^-ATPase, and basolateral K^+ ^channels [4,5]. This Na^+ ^absorption was blocked by amiloride and benzamil. The most commonly-observed target of these drugs is ENaC, comprised of the three subunits α-, β- and γ-ENaC. We addressed the question of cation selectivity of this pathway in Reissner's membrane epithelial cells with 5 series of patch clamp experiments and selective candidate gene expression analysis.

### Benzamil-sensitive currents under whole-cell patch clamp

We first tested whether benzamil-sensitive currents, which was earlier observed as transepithelial currents with the current-density vibrating probe [5], could be detected under whole-cell patch clamp conditions (Series 1). Indeed, benzamil (1 μM) reduced the inward current when the pipette and bath solutions (P1, B1) approximated the physiological situation (ignoring differences in apical cation and intracellular Cl^- ^composition) (Additional file 1: Fig. S1 and Fig. S3; Table 1).

**Table 1:** Inward and outward wholecell patch clamp currents, conductances and reversal voltage under established cationic conditions.

		I(-100) [pA]		g(-) [nS]		Vr [mV]		I(+100) [pA]		g(+) [nS]	
						
Series 1 Physiological	[Pipette]/[Bath]	Benz **(-)**	Benz **(+)**	Benz **(-)**	Benz **(+)**	Benz **(-)**	Benz **(+)**	Benz **(-)**	Benz **(+)**	Benz **(-)**	Benz **(+)**
						
	140KCl/150NaCl	-1305 {277 (7)}	-671 ‡ {146 (7)}	17.2 {2.1 (7)}	10.2 ‡ {1.1 (7)}	9.4 {3.7 (7)}	-2.0 ns {5.7 (7)}	938 {367 (7)}	708 ns {257 (7)}	9.9 {3.7 (7)}	7.2 ns {2.3 (7)}
Series 2 Na or K Bath	150Na-ms/150Na-ms	-2044 {552 (6)}	-255 ‡ {57 (6)}	25.9 {7.0 (6)}	2.6 ‡ {0.5 (6)}	0.5 {0.5 (6)}	-0.9 ns {1.0 (6)}	1496 {363 (6)}	769 ‡ {177 (6)}	16.5 {3.8 (6)}	13.9 ns {3.2 (6)}
						
	150K-ms/150K-ms	-476‡ {112 (8)}	-429 ns {130 (8)}	6.1 ‡ {1.3 (8)}	4.6 ns {1.3 (8)}	-3.8 ‡ {1.2 (8)}	-3.6 ns {1.2 (8)}	677 ns {218 (8)}	625 ns {218 (8)}	8.0 ns {2.5 (8)}	6.8 ns {2.4 (8)}

Series 3 Benzamil Na/Li	150Na-ms/150Na-ms	-1922 {96 (5)}	-639 ‡ {151 (5)}	19.8 {1.1 (5)}	5.9 ‡ {2.1 (5)}	0.7 {0.4 (5)}	-0.3 ‡ {0.5 (5)}	2109 {153 (5)}	1413 ‡ {165 (5)}	23.7 {1.6 (5)}	20.3 ‡ {1.7 (5)}
						
	150Na-ms/150Li-ms	-1656 {126 (5)}	-682 ‡ {86 (5)}	15.9 {1.3 (5)}	6.5 ‡ {0.7 (5)}	6.3 {1.5 (5)}	3.1 ‡ {1.5 (5)}	1619 {224 (5)}	1203 ‡ {144 (5)}	18.9 {2.6 (5)}	16.8 ns {1.8 (5)}

		Benz **(-)**		Benz **(-)**		Benz **(-)**		Benz **(-)**		Benz **(-)**	
						
Series 4 Na - Li	150Na-ms/150Na-ms	-1416 {181 (5)}		14.1 {1.6 (5)}		0.1 {0.4 (5)}		1710 {218 (5)}		18.3 {2.1 (5)}	
						
	150Na-ms/150Li-ms	-1699 ‡ {149 (5)}		16.2 ‡ {1.4 (5)}		7.1 ‡ {1.2 (5)}		1840 ns {148 (5)}		21.3 ns {1.5 (5)}	

		Benz **(-)**	Benz **(+)**	Benz **(-)**	Benz **(+)**	Benz **(-)**	Benz **(+)**	Benz **(-)**	Benz **(+)**	Benz **(-)**	Benz **(+)**
						
Series 5 All cations - benzamil	15Na-ms/150Na-ms	-3434 {363 (5)}	-741 ‡ {65 (5)}	31.2 {3.3 (5)}	6.1 ‡ {0.9 (5)}	29.8 {1.0 (5)}	21.1 ‡ {2.5 (5)}	1666 {243 (5)}	1039 ‡ {162 (5)}	25.7 {3.1 (5)}	21.1 ‡ {2.4 (5)}
						
		Benz **(-)**		Benz **(-)**		Benz **(-)**		Benz **(-)**		Benz **(-)**	
						
	15Na-ms/150NMDG-ms	-802 ‡ {118 (5)}		11.3‡ {1.4 (5)}		-27.8 ‡ {3.6 (5)}		1834 ns {342 (5)}		17.0 ‡ {3.0 (5)}	

Table entries are means on the top line and {SEM (number of experiments)} on the second line. Benz, benzamil; Benz(-), without benzamil; Benz(+), with benzamil (1 μM).

ms, Methanesulfonate, The number in front of chemical formula at [Pipette]/[Bath] is concentration (mM).

I(-100) and g(-), whole-cell patch clamp current and slope conductance at -100 mV command voltage; Vr, reversal voltage; I(+100) and g(+), current and conductance at +100 mV command voltage.

Significant differences: ‡, P < 0.05; ns, not significant.

Comparisons:

Series 1, 2, 3 and 5(Na ^+^)--Values in benzamil vs without benzamil (paired); Series 4--Values in Li^+ ^vs Na^+^; Series 5(NMDG^+^)-- NMDG^+ ^vs Na^+ ^(both in the absence of benzamil). In series 2, the symbols in the "Benz(+)" column report the significance of changes in values between ±benzamil using the paired t-test and the symbols in the "Benz(-)" column report the significance of changes in values between K^+ ^and Na^+ ^using the unpaired t-test.

Indeed, benzamil (1 μM) reduced the inward current when the KCl-rich pipette solution (P1) mimicked the presumed intracellular composition (although Cl^- ^was higher than often observed) and when the NaCl-rich bath (B1) mimicked the basolateral (perilymph) composition, (Additional file 1: Fig. S1 and Fig. S3; Table 1). Benzamil was used throughout these experiments at a concentration (1 μM) that yielded a complete inhibition of the transepithelial current [5] and significantly inhibited the inward whole-cell current (at -100 mV; I_-100_) by 48.5%; from -1305 ± 277 pA to -671 ± 146 pA (n = 7). These whole-cell data are consistent with the transepithelial measurements of Na^+ ^absorption by Reissner's membrane.

### Expression of benzamil-sensitive cation channels in RM

We utilized gene array and RT-PCR to partially address the question of the participation of benzamil-sensitive nonselective cation (NSC) channels. Several isoforms of acid-sensitive ion channels (ASIC) and cyclic-nucleotide gated (CNG) channels were listed in our gene array of mouse Reissner's membrane (GEO GSE6196; Table 2) [6]. ASIC1a, 2a, 2b, 3 and 4 were listed; ASIC1a and 3 yielded a call of "Present", but ASIC1a was tested by RT-PCR and determined to be "Absent" (Table 2). ASIC3 was not determined by RT-PCR; however, ASIC3 is stimulated, rather than inhibited, by amiloride at neutral pH [7] and therefore would not be expected to contribute to the currents measured in the present study. CNGA1, 2, 4 and CNGB3 were listed in the gene array and all received a call of "Absent". CNGA3 and CNGB1 were not listed in the gene array and were not tested by RT-PCR. As mentioned above, ENaC can be a NSC channel under some subunit combinations and the α-,β- and γ -ENaC subunits were all expressed in RM (Table 2).

**Table 2:** Transcript analysis of amiloride-sensitive channel genes in Reissner's membrane.

GenBank Accession No.	Gene	Protein	Call Gene array*	Call qRT-PCR*
AF112185	*Scnn1a*	αENaC	P	P
NM_011325	*Scnn1b*	βENaC	P	P
NM_011326	*Scnn1g*	γENaC	P	P
NM_009597	*Accn2*	ASIC1a	P**	A
NM_001034013	*Accn1*	ASIC2a	A**	A
NM_007384	*Accn2b*	ASIC2b	A**	A
NM_183000	*Accn3*	ASIC3	P	ND
NM_183022	*Accn4*	ASIC4	A	ND
NM_007723/U19717	*Cnga1*	CNGA1	A	ND
NM_007724	*Cnga2*	CNGA2	A	ND
NM_009918	*Cnga3*	CNGA3	NL	ND
NM_001033317	*Cnga4*	CNGA4	A	ND
HQ_116386	*Cngb1*	CNGB1	NL	ND
NM_013927	*Cngb3*	CNGB3	A	ND
NM_022017	*Trpv4*	TRPV4	P	P
NM_010408	*Hcn1*	HCN1	P	A
NM_008226	*Hcn2*	HCN2	P	A
NM_008227	*Hcn3*	HCN3	A	A
NM_001081192	*Hcn4*	HCN4	NL	A

*Call, gene was detected as present (P) or absent (A).

**Gene array annotation (Affymetrix #31) does not distinguish 'a' and 'b' variants. ND, Not Done; NL, Not Listed in gene array.

### Na^+ ^and K^+ ^conductance of benzamil-sensitive current

**Figure 1:**
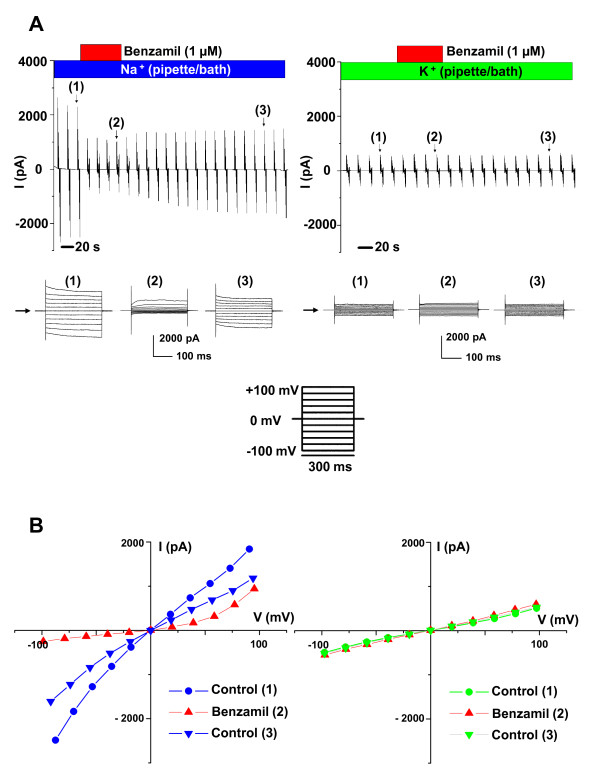
**Representative whole-cell patch clamp currents with only Na^+ ^or K^+ ^as the major permeant ions**. The compositions of the bath perfusates are indicated by the horizontal bars and the colors of the bars are identical to the colors in the corresponding graphs. *A) *Continuous trace recordings in symmetrical Na^+^-rich solutions (B2, P2; *left panel*) and K^+^-rich solutions (B3, P3; *right panel*). Voltage step protocols were applied every 16.7 seconds with holding at 0 mV and steps from +100 mV to -100 mV in 20 mV decrements. Individual step responses are shown in the lower panels and their locations within the continuous trace are indicated by the numbers in parentheses. Horizontal arrows indicate the zero current level. *B) *Current-voltage (I-V) relationships of the whole cell currents in the presence and absence of Benzamil (1 μM) at steady state (near 300 ms). Inhibition by benzamil was at least partially reversible and only observed in Na^+ ^solutions (*left panel*). K^+ ^currents (*right panel*) were markedly smaller than Na^+ ^currents.

**Figure 2:**
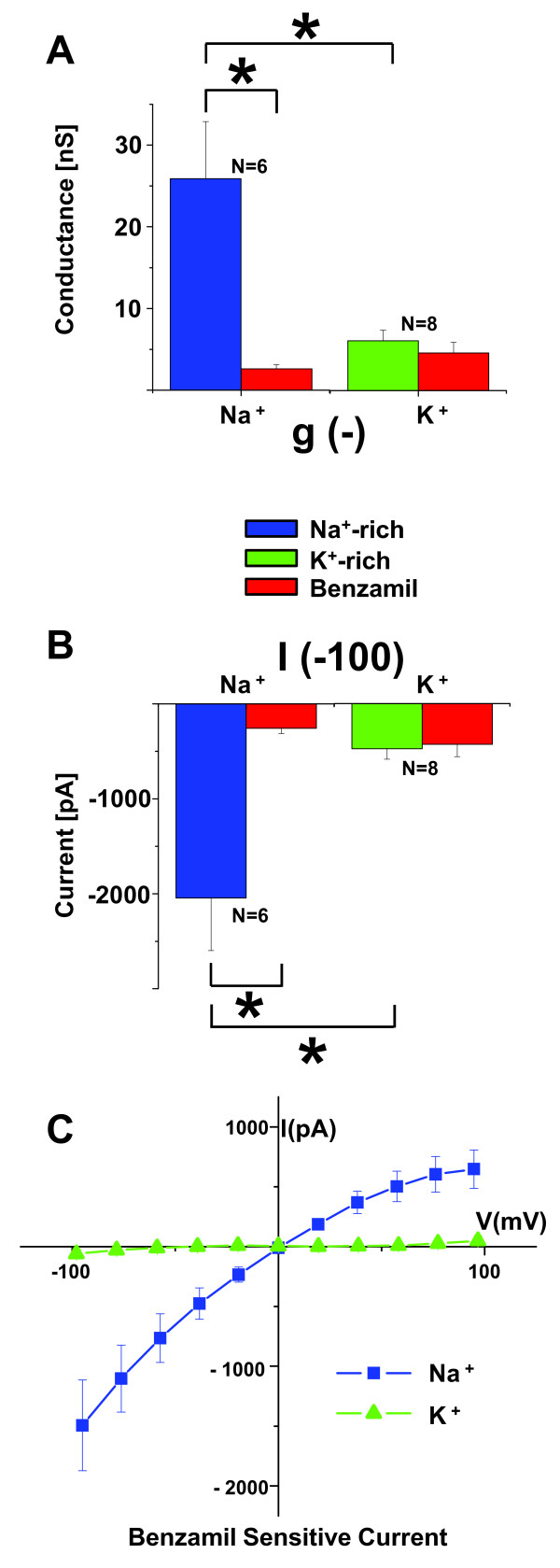
**Summary of whole-cell patch clamp currents with only Na^+ ^or K^+ ^as the major permeant ions**. *A) *Bar graph of the conductances and *B) *currents at -100 mV in symmetrical Na^+^-rich solutions (B2, P2) and K^+^-rich solutions (B3, P3). *C) *Current-voltage (I-V) relationships of the Benzamil-sensitive current at steady state (near 300 ms). Benzamil-sensitive current was only observed in Na^+ ^solutions. K^+ ^currents were markedly smaller than Na^+ ^currents.

Whole-cell patch clamp currents were measured (Series 2) under conditions where the only major permeant ions were either Na^+ ^or K^+ ^(Figure 1, 2). Cl^- ^was replaced by methanesulfonate (Table 3). In symmetrical Na^+^-rich bath and pipette solutions (B2, P2), the current-voltage (I-V) relationship was nearly linear, with a slight inward rectification at large negative voltage. The slight inward rectification observed at large negative voltage in the first series of symmetrical Na^+ ^experiments was at least partially due to the time dependence of the response. This series utilized 300 ms voltage steps and the second linear series utilized 200 ms steps. The strong rectification of the benzamil-sensitive current, however, may at least partially be due to the known voltage sensitivity of inhibition of ENaC by benzamil [8-10]. Benzamil significantly inhibited the inward whole-cell current (carried mostly by bath Na^+ ^at -100 mV) by 87.5%; from I_-100 _= -2044 ± 552 pA to -255 ± 57 pA (n = 6). Some experiments displayed a slow rundown in channel activity, as has been observed by others [8,11]. A representative experiment is shown in Figure 1 and a summary of similar experiments is shown in Figure 2 and Table 1.

**Table 3:** Bath and pipette solutions (mM)

	Bath					Pipette			
		
Solution	NaCl-rich	Na-rich	K-rich	Li-rich	NMDG-rich	KCl-rich	Na-rich	K-rich	NMDG+Na
	B1	B2	B3	B4	B5	P1	P2	P3	P4
NaCl	150					10			
KCl	4					140			
MgCl2	1					1			
CaCl2	0.7					0.273			
glucose	5	5	5	5	5				
HEPES	10	10	10	10	10	10	10	10	10
EGTA						1	1	1	1
Na-ms*		150					150		15
K-ms*			150					150	
Li-ms*				150					
NMDG-ms*					150				135
Ca-gluconate		0.7	0.7	0.7	0.7		0.367	0.367	0.367
MgSO4		1	1	1	1		1	1	1
adjusted by	NaOH	NaOH	KOH	LiOH	NMDG	KOH	NaOH	KOH	NMDG
pH	7.4	7.4	7.4	7.4	7.4	7.2	7.3	7.3	7.3

*ms: Methanesulfonate

In symmetrical K^+^-rich bath and pipette solutions (B3, P3), the current and conductance at -100 mV were significantly smaller than in Na^+ ^(Figure 1, 2; Table 1). Benzamil had no significant effect on the inward whole-cell current (carried mostly by bath K^+ ^at -100 mV) (mean decreased insignificantly by 9.8%; from I_-100 _= -476 ± 112 pA to -429 ± 130 pA, n = 8). A representative experiment is shown in Figure 1 and a summary of similar experiments is shown in Figure 2 and Table 1.

### Li^+^/Na^+ ^permeability ratio of benzamil-sensitive current

**Figure 3:**
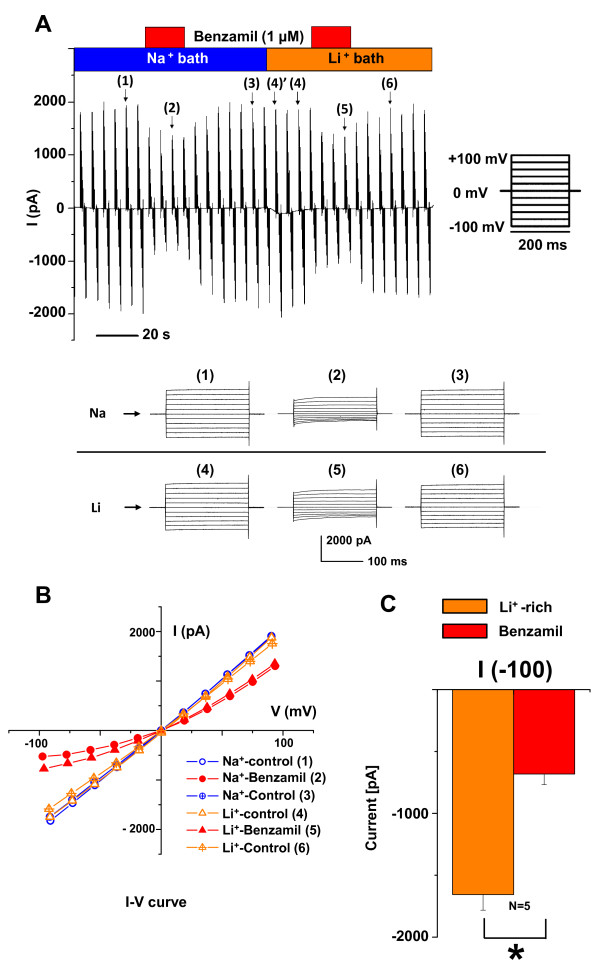
**Whole-cell patch clamp currents with only Na^+ ^or Li^+ ^as the major permeant ions**. The compositions of the bath perfusates are indicated by the horizontal bars and the colors of the bars are identical to the colors in the corresponding graphs. *A) *Representative continuous recording in symmetrical Na^+^-rich solutions (B2, P2; *left panel*) and Li^+^-rich solutions (B4, P2; *right panel*). Voltage step protocols were applied every 5.3 seconds with holding at 0 mV and steps from +100 mV to -100 mV in 20 mV decrements. Individual step responses are shown in the lower panels and their locations within the continuous trace are indicated by the numbers in parentheses. *B) *Representative current-voltage (I-V) relationships of the whole cell currents in the presence and absence of Benzamil (1 μM) at steady state (near 200 ms). The I-V relationship in Li^+ ^bath was qualitatively similar to that in Na^+ ^bath, as was the response to benzamil (1 μM).*C) *Summary bar graph of the currents at -100 mV in Li^+^-rich bath and Na^+^-rich pipette solutions (B4, P2).

The high Na^+ ^selectivity of the benzamil-sensitive current over K^+ ^suggested that ENaC might mediate part or all of that current. One salient characteristic of ENaC is a higher permeability to Li^+ ^than to Na^+^. We compared (Series 3, 4) whole-cell currents in the presence of Li^+ ^and Na^+ ^in the bath and the effects of benzamil. The comparisons were conducted in two series of paired measurements from the same experimental data set in order to minimize the effects of rundown. In the *first series of measurements *(Series 3), inward Li currents were tested for sensitivity to benzamil. A representative experiment is shown in Figure 3A and its I-V releationship in Figure 3B. The I-V relationships were very similar for both Na^+ ^(B2) and Li^+ ^(B4) and benzamil markedly decreased the inward currents for both ions. Specifically, benzamil significantly inhibited the inward Li^+ ^whole-cell current (at -100 mV) by 58.8%; from I_-100_ = -1656 ± 126 pA to -682 ± 86 pA (n = 5) (Figure 3B, C and Table 1). Similarly, benzamil decreased (Series 3) the inward Na^+ ^current at -100 mV by 66.8% (from I_-100_ = -1922 ± 96 pA to -639 ± 151 pA), a decrease that was not significantly different from the decrease by benzamil seen in the previous series (Series 2) of experiments with Na^+ ^in the bath (Figure 3B and Table 1).

It was to be expected that there would be no change in the observed V_r _between the presence and absence of benzamil since the reversal potential for both Na^+^-selectective and NSC channels are both = 0. By contrast, if Li^+ ^is more permeable than Na^+^, V_r _would be expected to become more positive (see second series, below).

**Figure 4:**
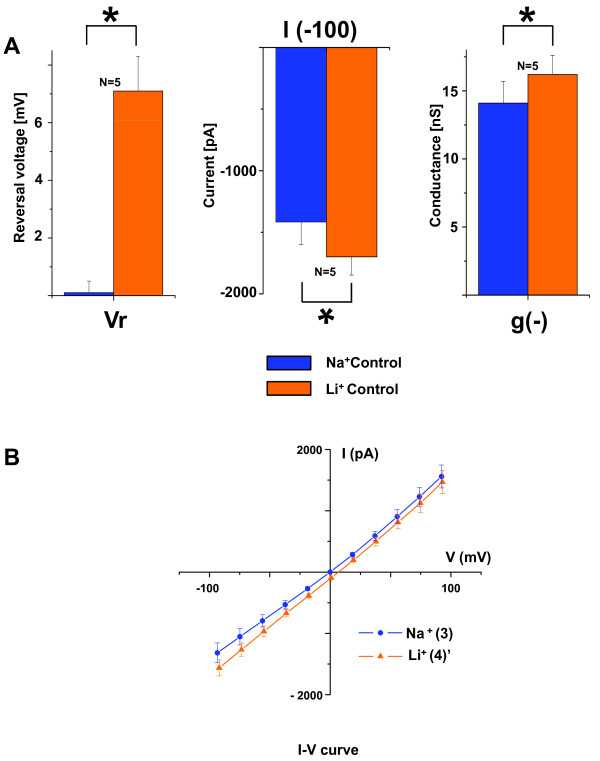
**Summary whole-cell patch clamp currents and reversal voltage with only Na^+ ^or Li^+ ^as the major permeant ions**. *A) *Bar graph of the reversal voltage (*left panel*), the currents at -100 mV (*middle panel*) and the conductance at -100 mV (right panel) in Na^+^-rich symmetrical solutions (B2, P2) and Li^+^-rich bath and Na^+^-rich pipette solutions (B4, P2). Data were taken from the rapid transition between Na^+^-rich and Li^+^-rich bath (time points 3 and 4' in Fig 3A) in order to minimize the contribution of rundown. *B) *Summary of current-voltage (I-V) relationships of the whole cell currents in symmetrical Na^+^-rich solutions (B2, P2) and Li^+^-rich bath and Na^+^-rich pipette solutions (B4, P2) at steady state (near 200 ms).

In the *second series of measurements *(Series 4), Na^+ ^and Li^+ ^I-V relationships were compared immediately before and after the solution change (points 3 and 4' in Figure 3A). Rapid change of the bath solution from a Na^+^-rich solution (B2) to a Li^+^-rich solution (B4) led to small but consistent changes in the current. The average inward current and conductance at large negative voltage (-100 mV) were significantly larger for Li^+ ^than for Na^+^, by 19.9% (I_-100_ = -1699 ±149 pA *vs *-1416 ±181 pA, n = 5) and 14.9% (g_-100_ = 16.2 ± 1.4 nS *vs *14.1 ± 1.6 nS, n = 5). Further, the reversal voltage was significantly more positive (7.1 ± 1.2 mV *vs *0.1 ± 0.4 mV) (Figure 4).

The shift in V_r _was analyzed with the Goldman-Hodgkin-Katz (GHK) equation as applied to bionic substitutions to estimate the relative membrane permeability to Li^+ ^and Na^+ ^[12]:

$$\Delta V_{r}=\frac{RT}{zF}In\frac{P_{Li}{\left[Li\right]}_{o}}{P_{Na}{\left[Na\right]}_{o}},$$

where ΔV_r _is the change in reversal potential, R the universal gas constant, T the absolute temperature, z the valence, F the Faraday constant, [Li]_o _the extracellular Li^+ ^concentration, [Na]_o _the extracellular Na^+ ^concentration and P_Li _and P_Na _the permeabilities of the membrane for Li^+ ^and Na^+^. The shift of V_r _by 7.0 mV in the current study corresponds to a P_Li_/P_Na _ratio of 1.3. This result is within the range of reports by others for the ratio in αβγ-ENaC (1.3 [13,14], 1.6 [11], 1.8 [15]).

### Benzamil-insensitive cation channels

We hypothesized that the residual current after inhibition by benzamil could be transported through nonselective cation channels, although a substantial contribution of NSC channels was unlikely since K^+ ^conductance was relatively small. Alternatively, the residual current could be a patch leakage current.

It was first observed that transcripts of TRPV4, an amiloride-insensitive NSC channel, were present by both gene array and RT-PCR (Table 2). The amiloride-insensitive channels HCN1-4 are permeable to K^+ ^and Na^+ ^(P_K_/P_Na _~4 [16]) and were found to be absent by RT-PCR, although some false positives in the gene array were found (Table 2).

We tested for the presence of benzamil-insensitive currents by comparing the inward current in the presence and absence of benzamil with a substitution of the impermeable cation NMDG^+ ^for Na^+ ^in the bath.

**Figure 5:**
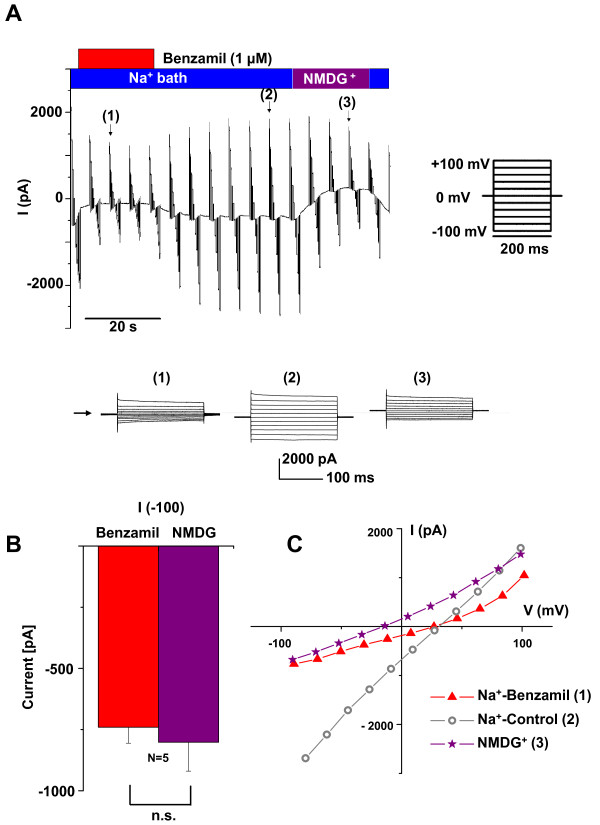
**Whole-cell patch clamp currents with only Na^+ ^or NMDG^+ ^as the major permeant ions**. The compositions of the bath perfusates are indicated by the horizontal bars and the colors of the bars are identical to the colors in the corresponding graphs. *A) *Representative continuous trace recordings in the presence and absence of Benzamil (1 μM) in Na^+^-rich bath and 15 mM Na^+ ^pipette solutions (B2, P4) and NMDG^+^-rich bath and 15 mM Na^+ ^pipette solutions (B5, P4). Voltage step protocols were applied every 5.3 seconds with holding at 0 mV and steps from +100 mV to -100 mV in 20 mV decrements. Individual step responses are shown in the lower panels and their locations within the continuous trace are indicated by the numbers in parentheses. *B) *Summary bar graph of the currents at -100 mV in Na^+^-rich bath (B2) with Benzamil (1 μM) and NMDG^+^-rich bath solutions (B5). *C) *Representative current-voltage (I-V) relationships of the whole cell currents in the presence and absence of Benzamil (1 μM) in Na^+^-rich bath and 15 mM Na^+ ^pipette solutions (B2, P4) and NMDG^+^-rich bath and 15 mM Na^+ ^pipette solutions (B5, P4) at steady state (near 200 ms). NMDG^+ ^reduced the inward current (-100 mV) by the same amount as did benzamil, and there was no difference between outward current (+100 mV) in NMDG^+^- and Na^+^-rich baths (B5 and B2)

In this series of experiments (Series 5), the initial phase before benzamil did not quickly reach a stable condition so that parameters in benzamil were compared to those after washout. Nonetheless, it is clear that the inward current and conductance in benzamil were significantly less than the inward Na^+ ^current and conductance after washout (Figure 5, Table 1). Importantly, there was no significant difference between the inward current in benzamil (I_-100_ = -741 ± 65 pA, n = 5) and in NMDG^+ ^(I_-100_ = -802 ± 118 pA, n = 5, B5). These results demonstrate that there were no cation currents other than the benzamil-sensitive channels under these conditions.

Na^+ ^in the pipette was reduced to 15 mM (P4) in order to confirm the Na^+^-selectivity of the current in this experimental series. The large decrease in V_r _between Na^+ ^(B2) and NMDG^+ ^(B5) (from 29.8 ± 1.0 mV to -27.8 ± 3.6 mV, n = 5; Figure 5 and Table 1) is consistent with a large Na^+ ^conductance. The small, but significant, decrease in V_r _between ± benzamil could conceivably be due to the presence of an H^+ ^conductance and the small pH gradient between intra- and extracellular solutions. The equilibrium potential for Na^+ ^was +60 mV and for H^+ ^was +6 mV. The absence of any significant difference (Table 1) in I_+100 _(14834 ± 342 pA *vs *1666 ± 243 pA, n = 5) in NMDG^+ ^and Na^+ ^baths (B5 and B2) is consistent with the current at +100 mV being carried predominantly by the efflux of intracellular Na^+^, since the pipette concentration (P4) remained the same for both bath solutions.

## Discussion

Active Na^+ ^absorption is needed to remove Na^+ ^that has entered cochlear endolymph through as-yet-undefined pathways. Elevated Na^+ ^would provide an osmotic driving force for water entry that would lead to endolymphatic hydrops, a pathologic swelling of the luminal space. In addition, the cochlear epithelium requires parasensory K^+^-efflux pathways to compensate for changes in K^+ ^efflux through sensory hair cells during changes in auditory stimulation.

In addition to Na^+ ^and K^+ ^absorption from endolymph via the outer sulcus cells in the cochlear lateral wall [17], Reissner's membrane epithelium has been found to actively absorb Na^+ ^from endolymph via amiloride- and benzamil-sensitive pathways. This transport was thought to be mediated by the epithelial Na^+ ^channel (ENaC) in the apical membrane of these cells [4,5].

The classical ENaC is highly selective to the passage of Na^+ ^over K^+ ^(P_Na_/P_K _> 80). However, there are reports of poorly-selective epithelial Na^+ ^absorptive pathways under some culture conditions and this can be due to either altered ENaC subunit stoichiometry or increased expression of unrelated nonselective cation channels [18]. Heteromeric channels composed of the α-, β- and γ-ENaC subunits are highly Na^+^-selective, while homomeric α-ENaC channels are also permeable to K^+ ^[18]. Homo- and hetero-meric ENaC are pharmacologically defined by their inhibition by amiloride and its analogs [19]. All 3 ENaC subunits are expressed in Reissner's membrane [5], but the stoichiometric subunit association in the apical membrane of Reissner's membrane epithelial cells is not known. In addition, other cation channels such as acid sensitive ion channels (ASIC) and cyclic nucleotide gated channels (CNG) have been observed to be sensitive to amiloride and benzamil (micromolar to submicromolar range) [20,21].

A vestige of doubt therefore remained whether the benzamil-sensitive current could also carry K^+ ^as well as Na^+^.

We tested the hypothesis that the benzamil-sensitive Na^+ ^absorption pathway in Reissner's membrane could also carry a K^+ ^current. Our results showed the absence of transcript expression of many candidate nonselective cation channels and the functional absence of both benzamil-sensitive and -insensitve nonselective cation currents. The results demonstrated a high selectivity of the benzamil-sensitive pathway to Na^+ ^over K^+^.

One further test of the molecular identity of the benzamil-sensitive pathway was a comparison of the permeability to Li^+ ^and Na^+^. The greater permeability to Li^+ ^is consistent with the benzamil-sensitive current carried by the classical αβγ -ENaC.

## Conclusions

**Figure 6:**
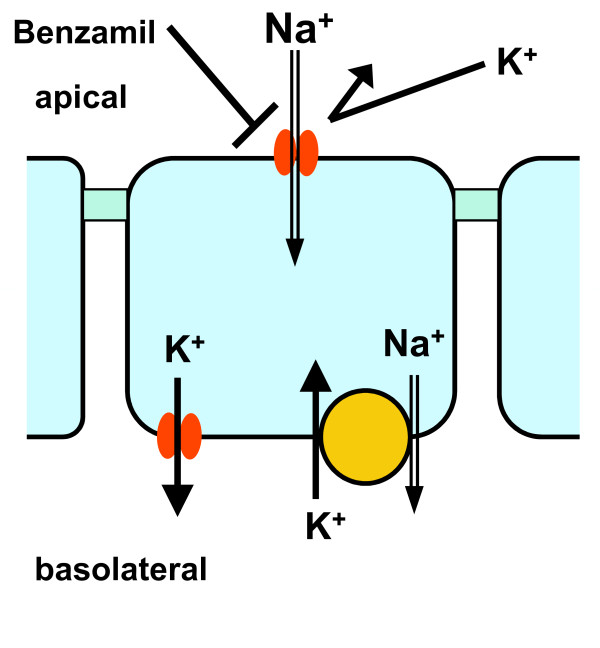
**Na^+^-selective transepithelial transport by Reissner's membrane**. Na^+ ^from endolymph enters the cell across the apical membrane through an amiloride- and benzamil-sensitive cation channel that excludes K^+^. Na^+ ^in the cytosol is extruded across the basolateral membrane into perilymph of scala vestibuli by the Na^+^-pump (Na^+^, K^+^-ATPase). K^+ ^entering the cell by the pump is passively extruded across the basolateral membrane via K^+ ^channels.

The results of this study support the conclusion that the benzamil-sensitive whole-cell current in Reissner's membrane epithelial cells is highly selective for Na^+ ^and does not support the transport of K^+^. When this is combined with the results of previous studies on the benzamil-sensitive transepithelial current, it suggests that Reissner's membrane contributes to maintaining the low Na^+ ^concentration in normal endolymph but is not involved in K^+ ^homeostasis through this pathway (Figure 6).

## Methods

### Tissue preparation for electrophysiological recordings

Tissue was obtained, prepared and analyzed as described previously [22]. Mice (C57BL/6) were anesthetized with 4% tribromoethanol (0.014 ml/g body wt ip) and killed following a protocol approved by the Institutional Animal Care and Use Committee of Kansas State University. The lateral wall of the cochlear apical turn was isolated from the temporal bone, the stria vascularis removed and Reissner's membrane was gently pushed into the same plane as the spiral ligament. Reissner's membrane consists of an epithelial cell layer facing the endolymph and a mesothelial cell layer facing the perilymph, the two layers being separated by a basement membrane. The tissue was folded with the apical membrane of the epithelium on the exterior of the loop in order to form patch clamp seals with the apical membrane (Additional file 1: Fig. S3).

### Whole cell patch clamp recording

Currents were recorded using the whole-cell configuration of the patch clamp technique, similar to our previous study [22]. Patch pipettes were made from borosilicate glass capillaries (1B150F; World Precision Instruments, Sarasota, FL), pulled in three stages with an electrode puller (Narishige, East Meadow, NY). Inner diameter of the tip was approximately 2 μm and after heat polishing the pipettes had resistances of 3.3 - 9.6 MΩ (n = 31) in the different bath solutions.

Currents were recorded with an Axopatch 200A amplifier (Axon Instruments, Foster City, CA) and low-pass filtered at 1 kHz. Current signals were digitized at 5 kHz using a computer with a Digidata 1322A (Axon Instruments) and pCLAMP 9 software (clampex9, Axon Instruments) was used for voltage step protocol recordings. In addition, AxoScope software (Axon Instruments) with MiniDigi 1A (Axon Instruments) data acquisition hardware was simultaneously used for continuous trace recordings and current signals were digitized at 1 kHz. The temperature was maintained at 37°C on a glass-bottomed bath chamber by a continuous, warmed perfusion and supplemental chamber heater.

For the pipette solution containing high K^+ ^(P3) and bath containing high Na^+ ^(B2), the data were corrected for the liquid junction potential of 4.1 mV. For the pipette solution containing low Na^+ ^and high NMDG^+ ^(P4) and baths containing high Na^+ ^(B2) and high NMDG^+ ^(B5), the data were corrected for the liquid junction potentials of -6.9 mV and -6.2 mV (the latter includes the liquid junction potential change of 0.7 mV at the reference electrode). Liquid junction potentials in symmetric Na-Methanesulfonate, K-Methanesulfonate, NMDG-Methanesulfonate were near zero, since voltage offset was adjusted when the pipette was immersed in each bath solution. The Li^+ ^data were corrected by adding the 0.3 mV junction potential change at the reference electrode.

Two voltage protocols were used. *Protocol 1: *A series of pulses were applied for 300 ms each from a holding potential of 0 mV to steps from +100 to -100 mV in 20 mV decrements. The protocol was repeated every 16.7 s. Current-voltage (I-V) relationships were determined from averaging 30 ms of steady-state currents near the end of each voltage step. *Protocol 2: *This series only differed from the first protocol by the step duration (200 mS) and the repetition rate (5.3 s). The I-V relationships were obtained from these current averages plotted against the command voltage, corrected for the liquid junction potential and an estimate of the voltage drop across the pipette resistance. These corrected curves were used to obtain the currents and conductances at ±100 mV (I_-100_, I_+100_, g_+100_ and g_-100_) and the reversal voltage, V_r _(Additional file 1: Fig. S4). Data were plotted with Origin software, version 7 (OriginLab Software, Northampton, MA).

### Solutions and chemicals

Table 3 shows the composition of the solutions of pipette and bath for electrophysiological recordings. The osmolarity of these solutions was about 311-318 mOsm. Benzamil (Sigma #B-2417) was predissolved (1 mM) in dimethylsulfoxide (DMSO), and was used at a final concentration of 1 μM benzamil and 0.1% DMSO. Pipette solutions (P1, P2, P3, P4) were buffered to 100 nM free Ca^2+ ^[23]. The KCl-rich pipette solution (P1) was adjusted to pH 7.3 at room temperature, which corresponds to about 7.2 at 37°C and alters the Ca^2+^/EGTA ratio at that elevated temperature. Pipettes using P2, P3 and P4 were backfilled with 15 mM Cl^- ^substituted for methanesulfonate in order to make stable contact with the Ag/AgCl electrode. The backfill solutions contained the dye fast green to visualize the interface with the tip solution, which filled the first 10 mm of the pipette. All pipette solutions for patch clamp were passed through 0.22 μm cellulose acetate filters (Corning).

### RNA isolation for RT-PCR

Four mice (C57BL/6, 4-10 weeks old) were anesthetized with 4% tribromoethanol (0.014 ml/g body wt ip) and killed for each collection of total RNA. The two temporal bones were removed from each mouse after a transcardial perfusion of a phosphate-buffered saline (PBS) solution to reduce both cell swelling and contamination from blood cells. Reissner's membrane was dissected in PBS, which was changed twice during isolation of Reissner's membrane to reduce cross contamination from other tissues. All procedures were approved by the Institutional Animal Care and Use Committee of Kansas State University. Total RNA was isolated from the tissue using an RNeasy Micro Kit with carrier RNA (Qiagen, Valencia, CA). RNA used as positive control was from mouse kidney, mouse brain (Ambion, Austin, TX). The quality and quantity of RNA were determined with a Bioanalyzer (Additional file 1: Fig. S3B; Agilent, Palo Alto, CA). RT-PCR was conducted as described before [22].

### Statistical analysis

Data were expressed as the mean ± S.E.M. (n = number of whole cell patches). Increases and decreases in current and conductance were determined by Student's paired or unpaired t-test as appropriate for the specific data sets. Differences were considered statistically significant at a level of P < 0.05 (Microsoft Excel).

## Authors' contributions

MY carried out the study on Reissner's membrane, including microdissection of tissues and patch clamp recordings, and contributed to writing the manuscript. KXK carried out the study on Reissner's membrane, including microdissection of tissues, primer design and validation, RNA isolation, quantitative analyses of PCR, preliminary patch clamp recordings and contributed to writing the manuscript. DCM conceived of the study, and participated in its design and coordination and contributed to writing the manuscript. All authors have read and approved the final manuscript.

## Authors' information

MY is a clinician/researcher in the Dept. of Otolaryngology-Head and Neck Surgery at Tohoku University who conducted this research in partial fulfillment of requirements for the PhD degree at the Tohoku University Graduate School of Medicine. KXK contributed to this work while a candidate for a Masters degree at Kansas State University and upon completion of the requirements for the Master of Science degree, she has become a doctoral candidate in the Neuroscience Training Program at the University of Wisconsin. DCM is a University Distinguished Professor at Kansas State University in the Dept. of Anatomy & Physiology and heads the Cellular Biophysics Laboratory.

## Supplementary Material

Additional file 1 **Fig. S1, S2, S3, S4**. Benzamil-sensitive whole cell patch clamp currents under quasi-physiologic conditions (representative experiment and data summary), illustration of the patch clamp preparation and the quality of total RNA collected from Reissner's membrane, and illustrative description of the correction of command voltages in I-V plots. Click here for file
