# Supplementary material for: Sodium selectivity of Reissner's membrane epithelial cells
Source: BMC Physiol. 2011 Feb 1;11:4. doi: 10.1186/1472-6793-11-4 (PMC3042420; doi:10.1186/1472-6793-11-4)
Supplement: Additional file 1 — Fig. S1, S2, S3, S4. Benzamil-sensitive whole cell patch clamp currents under quasi-physiologic conditions (representative experiment and data summary), illustration of the patch clamp preparation and the quality of total RNA collected from Reissner's membrane, and illustrative description of the correction of command voltages in I-V plots. [file 1472-6793-11-4-S1.PDF]

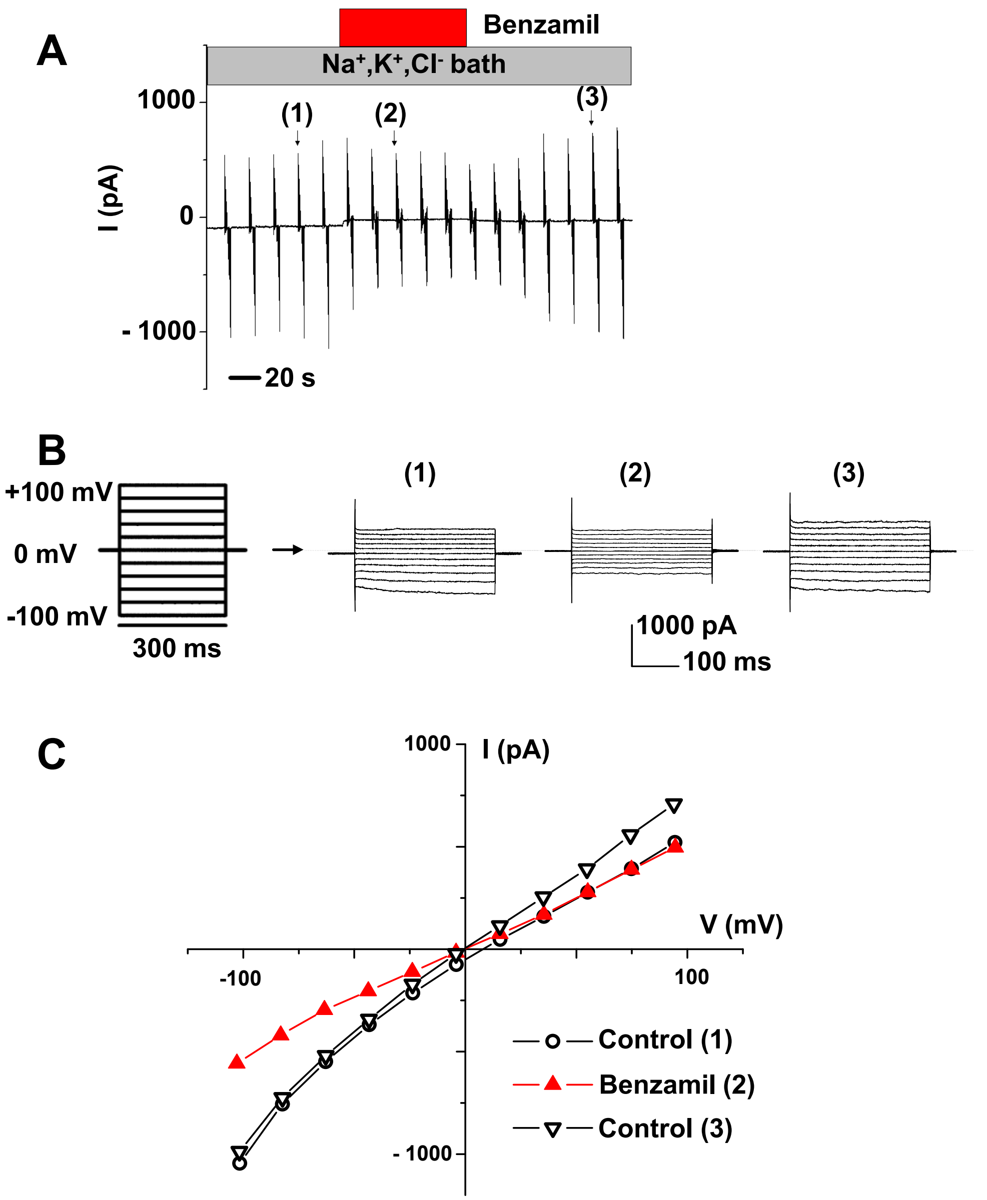

Fig. S1 - Representative whole-cell patch clamp currents with the KCl-rich pipette solution and the NaCl-rich bath solution.

The compositions of the bath perfusates are indicated by the horizontal bars and the colors of the bars are identical to the colors in the corresponding graphs. ***A)*** Continuous trace recording with the KCl-rich pipette solution (P1) and with the NaCl-rich bath (B1) that approximated the physiological situation. ***B)*** A voltage step protocol was applied every 16.7 seconds with holding at 0 mV and steps from +100 mV to -100 mV in 20 mV decrements. Individual step responses are shown in the right panels and their locations within the continuous trace are indicated by the numbers in parentheses. ***C)*** Current-voltage (I-V) relationships of the whole cell currents in the presence and absence of Benzamil (1μM) at steady state (near 300 ms). Benzamil reduced the inward current.

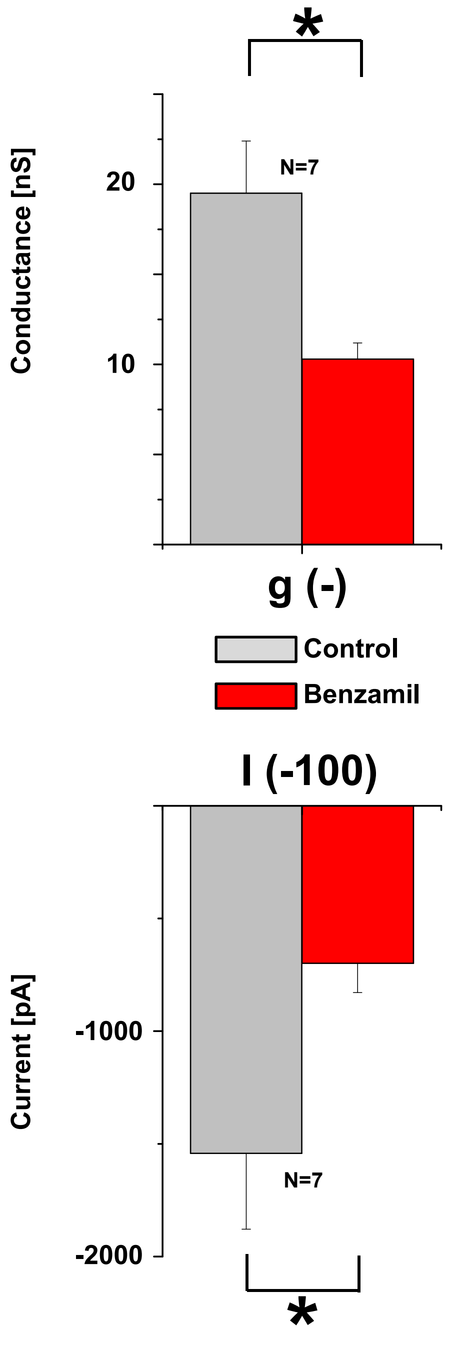

Fig. S2 - Summary of whole-cell patch clamp currents with the KCl-rich pipette solution and the NaCl-rich bath solution.

Bar graphs of the conductances (*upper panel*) and currents (*lower panel*) at -100 mV summarized from experiments as illustrated in Figure S1.

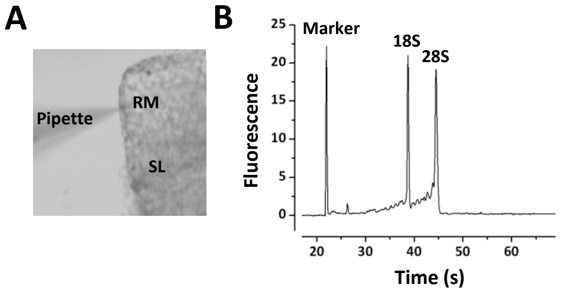

Fig. S3 - Tissue preparation for Reissner's membrane and quality of isolated RNA. ***A)*** Photomicrograph of the folded preparation of Reissner's membrane (RM) with patch pipette electrode. SL, spiral ligament. ***B)*** Representative electropherogram showing high quality of total RNA obtained from Reissner's membrane. Sharp peaks representing 18S and 28S rRNA and low background demonstrate high quality of RNA.

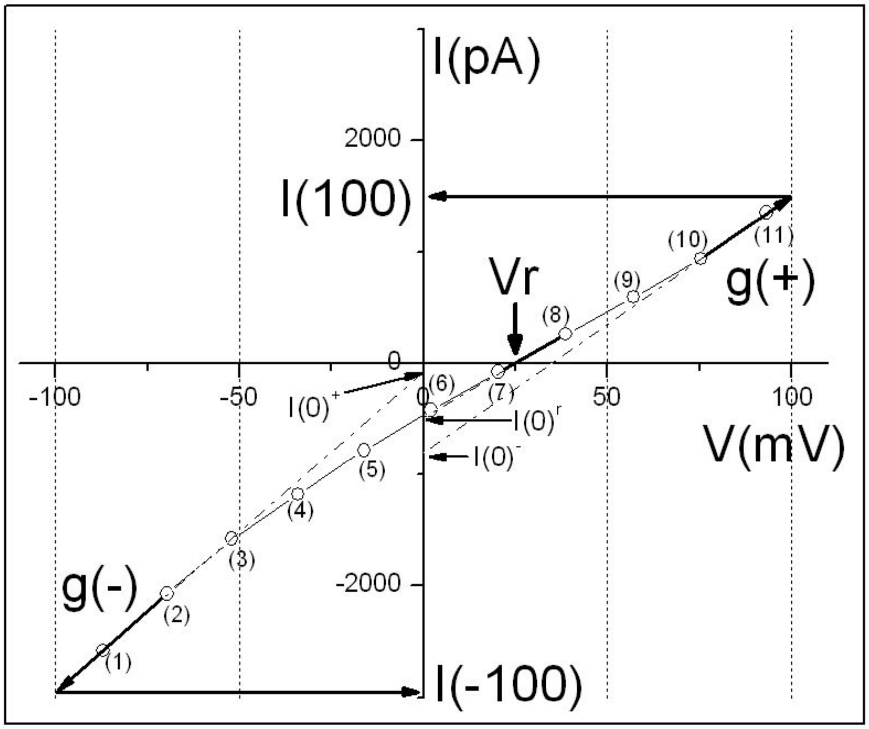

Figure S4 - Calculation of conductances, currents and reversal voltage

Command voltage offsets were corrected for liquid junction potentials and voltage drops across the series resistance of the patch electrode. We solved the linear equation [I(+100)=g(+)·V+I(0)+] from the most positive two points (points 10 and 11 in the figure) and calculated the whole-cell patch clamp current [I(100)] and slope conductance [g(+)] at (corrected) +100 mV computer command voltage. Similarly, the corresponding currents and conductances at (corrected) -100 mV were derived from the equation [I(-100)=g(-)·V+I(0)-] using the most negative two points (points 1 and 2 in the figure). Similarly, we derived the reversal voltage, V_r, using the equation [I(0)=g(0)·V+I(0)r] using the two points closest to zero current (points 7 and 8 in the figure).
